# Efficacy of laser acupuncture for patients with chronic Bell's palsy: A study protocol for a randomized, double-blind, sham-controlled pilot trial

**DOI:** 10.1097/MD.0000000000015120

**Published:** 2019-04-12

**Authors:** Gil Ton, Li-Wen Lee, Hui-Ping Ng, Hsien-Yin Liao, Yi- Hung Chen, Cheng-Hao Tu, Chun-Hung Tseng, Wen-Chao Ho, Yu-Chen Lee

**Affiliations:** a Graduate Institute of Acupuncture Science; b Department of Public Health; c Department of Neurology; d Department of Acupuncture; e International Master Program in Acupuncture; f Chinese Medicine Research Center, China Medical University, Taichung, Taiwan.

**Keywords:** laser acupuncture, low-level laser therapy, randomized controlled trial, sequelae of Bell's palsy

## Abstract

**Background:**

Bell's palsy is the most frequent cause of unilateral peripheral facial palsy, a common condition that third of patients can have inadequate recovery and subsequent physical and social impairments. The largely ineffective and even controversial nature of the various medical and surgical treatment options means that novel, alternative approaches are needed. In preclinical and clinical evidence, low-level laser therapy (LLLT) has demonstrated the ability to regenerate peripheral nerves. Laser acupuncture treatment (LAT), the stimulation of traditional acupoints with low-intensity, non-thermal laser irradiation, is a common treatment modality, but its efficacy in chronic Bell's palsy is undetermined. This study aims to evaluate the efficacy of LAT in patients experiencing inadequate recovery from Bell's palsy.

**Methods:**

This 2-armed, parallel, randomized, subject-assessor-blinded, single-center, sham-controlled pilot trial will randomly assign 32 eligible patients into either a real LAT group (n = 16) or a sham LAT group (n = 16). The real LAT group will receive 3 LAT sessions each week for 6 weeks (a total of 18 sessions), delivered to acupoints corresponding with the affected side of the face. The sham LAT group will receive the same treatment as the real LAT group, but with a sham laser device. The primary outcome measure will be the change from baseline at week 6 in the Facial Disability Index score. Secondary outcomes will monitor changes during treatment in the House-Brackmann and Sunnybrook facial nerve grading systems and stiffness scale, at weeks 1, 3, and 6.

**Discussion:**

To the best of our knowledge, this double-blind, randomized, sham-controlled trial is the first such investigation into the efficacy of LAT in chronic Bell's palsy. Clinical trials using LLLT have shown positive therapeutic effects in acute Bell's palsy, although as yet, the feasibility and efficacy of LAT remain unclear in patients experiencing inadequate recovery from Bell's palsy.

**Trial registration:**

This trial protocol has been approved by the Research Ethics Committee of the China Medical University Hospital, Taichung, Taiwan (Protocol ID: CMUH107-REC1-030) also registered at ClinicalTrials.gov (identifier no. NCT03592797).

## 1 Introduction

The human face is unique and an extremely important aspect of a person's identity. Facial expressions play a significant role in social interactions, as humans express their emotions through use of facial muscles. Thus, besides the loss of functional ability, any impairment in facial muscle control creates considerable social distress.^[1]^

Bell's palsy is the most frequent cause of unilateral peripheral facial palsy.^[2]^ This form of paralysis is especially common between the ages of 15 and 45 years; in the USA, 20 to 30 cases per 100,000 population are diagnosed annually.^[3]^ Symptom onset is typically sudden, ranging in severity from mild to severe and often peaking within 24 to 72 hours. Symptoms include facial muscular weakness, retroauricular pain, impaired tolerance of noise and ipsilateral disturbance of taste.^[4]^ Overall, the prognosis is favorable. In 1 study involving 1701 patients with Bell's palsy, 1202 (71%) recovered normal facial function without any intervention.^[4]^ Of the 1189 patients with complete paralysis, 721 (61%) regained normal mimical function; the reminder did not. Notably, the likelihood of regaining normal function was low after 3 months, at which time 64% of patients had regained normal function; none of the patients who still experienced functional problems beyond 6 months regained normal mimical function.^[4]^ Long-term sequelae in Bell's palsy is defined as facial palsy persisting beyond 3 months after the onset of symptoms.^[5]^

Incomplete recovery from Bell's palsy results in physical and social impairments. Cases of persistent facial paralysis without progressive symptomatic improvement can consider the use of invasive treatments like surgical decompression or botulinum toxin injection, although controversy surrounds the benefits associated with these options and others.^[5,6]^ In Asia, acupuncture is widely used for the management of Bell's palsy. A number of studies, especially those from China, have reported on average a recovery rate of 80% and benefits of acupuncture in cases of acute Bell's palsy.^[6]^ In a recent meta-analysis, acupuncture was associated with an effective response rate for Bell's palsy,^[7]^ while other research has beneficial effects of acupuncture for the sequelae of Bell's palsy.^[8]^ According to this study, acupuncture improved facial nerve function and reduced the patients’ social impairments.

Acupuncture has become one of the most common alternative treatment options in Western countries.^[9]^ Its reputation for good efficacy minimal side effects in a myriad of therapeutic conditions has fostered a rapid growth in acupuncture research and practice^[10]^ and the development of other forms of acupuncture besides the conventional stainless steel acupuncture needles, such as laser acupuncture treatment (LAT), defined as the stimulation of traditional acupuncture points with low-intensity, non-thermal laser irradiation.^[10]^ Notably, low-level laser therapy (LLLT) has shown beneficial outcome in the regeneration of peripheral nerves^[11,12]^ and several clinical studies have shown that LLLT is effective for treating acute Bell's palsy. However, none of these laser treatment studies used acupuncture points as the irradiation target sites or investigated its effectiveness in chronic Bell's palsy.^[13,14]^ This pilot study was therefore designed to determine the feasibility and efficacy of LAT in patients experiencing inadequate recovery from Bell's palsy.

## 2 Methods/design

### 2.1 Objective

The primary objective of this pilot study is to assess the feasibility and therapeutic efficacy of LAT in patients experiencing inadequate recovery from Bell's palsy.

### 2.2 Design

**Figure 1:**
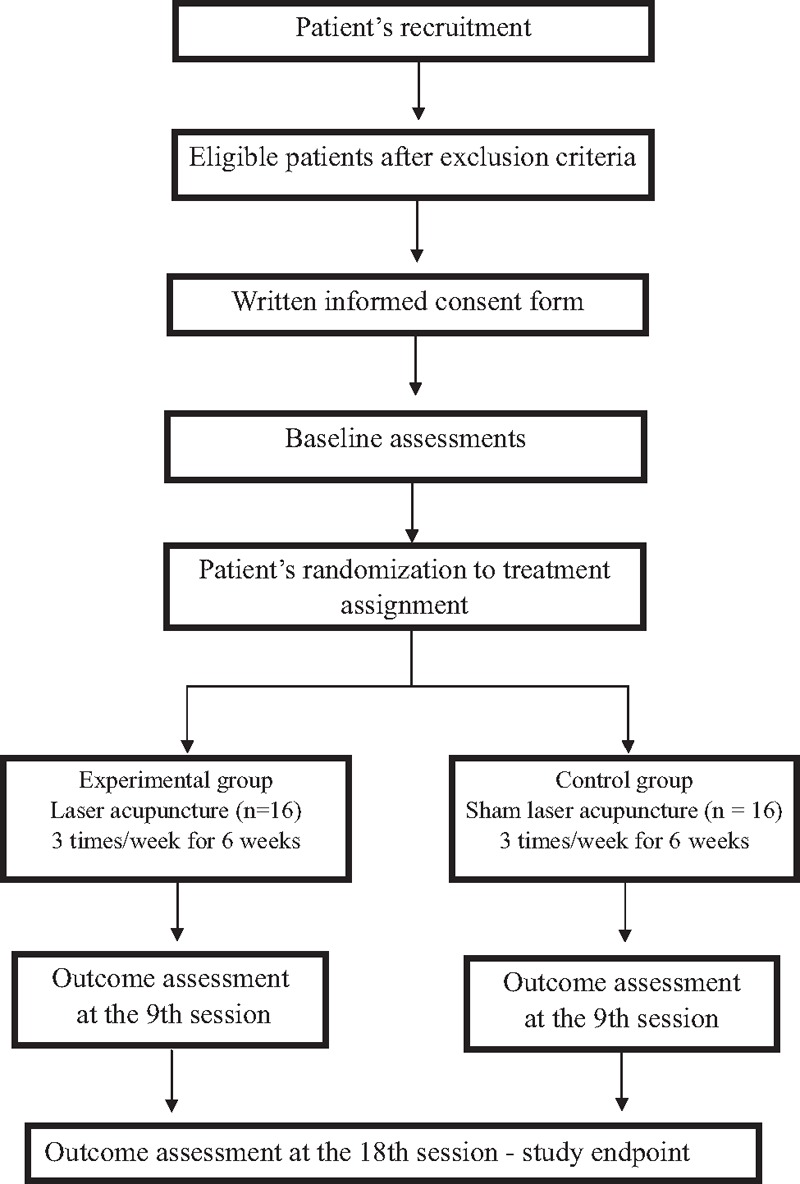
Study design flow chart.

A double-blind, randomized, sham-controlled clinical study will be conducted in the Acupuncture Department of China Medical University Hospital in Taichung, Taiwan. The study flowchart and assessment schedule are shown in Figure 1 and Table 1, respectively. In order to ensure that the assessments of patients will not be affected by knowledge of treatment assignment, the investigator and patients will be blinded to the procedure. A normal laser device will be used in the real LAT group and a sham laser device in the sham LAT group. The normal laser device will be equipped with red laser light for the purpose of locating the point and acoustic signaling informing the user as to when the infrared irradiation begins and ends. The laser irradiation will be deactivated in the sham laser device by its manufacturer (RJ laser, Germany), but its visual light and acoustic functions maintained. The output power of the sham laser's infrared light will be zero mW, as according to the manufacturer. This kind of sham laser procedure has shown good reliability and validity.^[15]^ The study will follow the Standard Protocol Items Recommendations for Interventional Trials (SPIRIT) 2013 statement^[16]^ and the 2010 checklist detailed in the Standards for Reporting Interventions in Clinical Trials of Acupuncture^[17]^ (STRICTA) (Table 2).

**Table 1:**
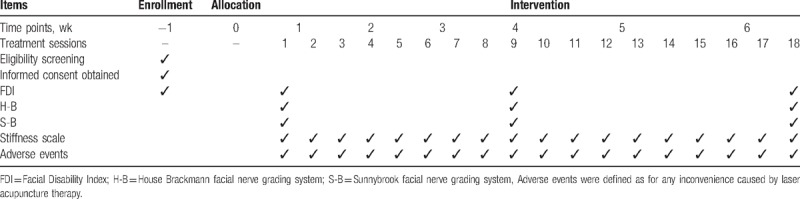
The schedule of enrolment, interventions, and assessments.

**Table 2:**
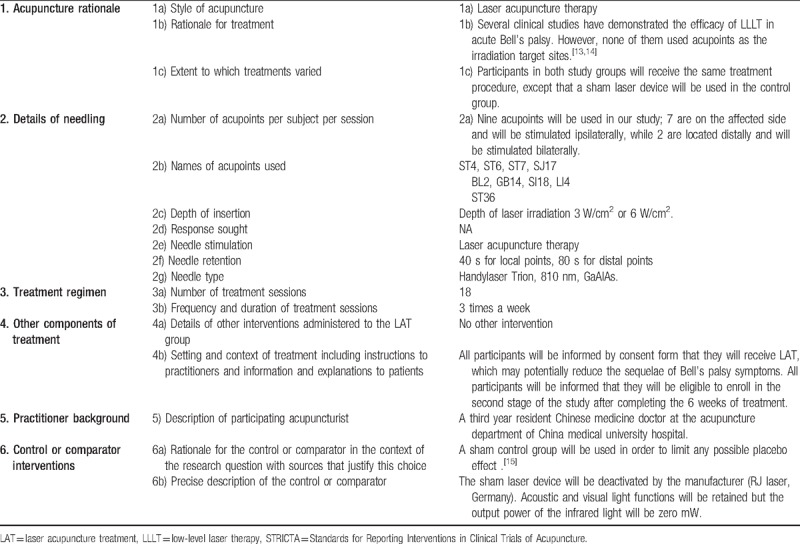
The laser acupuncture treatment protocol will follow the STRICTA 2010 checklist^[17]^.

### 2.3 Recruitment of participants

**Figure 2:**
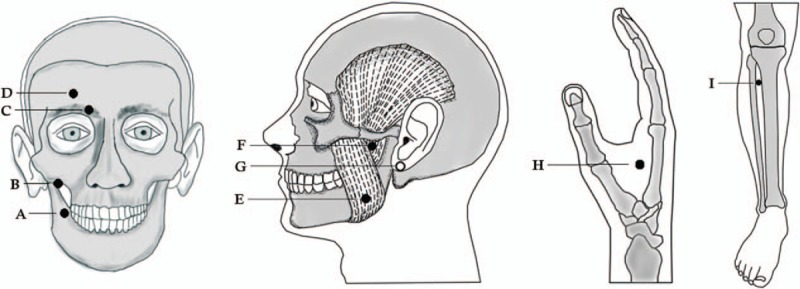
Acupoint used for chronic Bell's palsy: **A** ST4 **B** SI18 **C** BL2 **D** GB14 **E** ST6 **F** ST7 **G** SJ17 **H** LI4 **I** ST36.

A total of 32 patients experiencing inadequate recovery from Bell's palsy will be recruited through advertisement via posters in the clinics of the Acupuncture and Neurology departments, China Medical University Hospital, Taichung, Taiwan. The recruitment period will be from May 2018 and expected to end in May 2020. The screening process will be conducted by the chief doctor of the Acupuncture department who will verify the patient's condition and hand-in the Facial Disability Index (FDI) for the patient. If the condition will match, the research assistant will contact the patient and schedule an appointment, in which the patient will be explained all the conditions of the research and sign the written consent form. After that, the first session will be held in which the patient's condition will be assessed and the patient will get a treatment from the study investigator. Our baseline assessments include the FDI scoring system, the House-Brackmann (H-B) facial nerve grading system, the Sunnybrook (S-B) facial nerve grading system, and stiffness scale. Eligible participants will be randomized to 1 of 2 groups: the real LAT group or the sham LAT group; all will receive LAT treatments 3 times per week for a total of 18 sessions over 6 weeks. Both groups will receive treatment at 9 acupoints (ST4, ST6, ST7, SI18, BL2, GB14, and SJ17 on the affected side, ST36 and LI4 bilaterally) in each acupuncture session (Fig. 2), administered by an experienced traditional Chinese medicine physician. Whereas the real LAT group will receive LAT treatment with a true laser device, the sham LAT group will receive LAT delivered by a sham device with a deactivated laser. At the end of the 6 weeks of treatment, the sham LAT group will be offered to receive a real laser treatment similarly to the LAT group. The second phase sessions for the sham group will be administered by a second investigator.

### 2.4 Inclusion criteria

The study will enroll patients with a diagnosis of Bell's palsy (ICD-9-CM Diagnostic Code 351.0) who meet the following inclusion criteria:

(a) FDI scores of <80 on the physical and social subscales;
(b) age >20 years;
(c) male or female gender;
(d) symptoms of right-or-left side-sided Bell's palsy for ≥3 months before screening;
(e) able and willing to comply with the intervention and follow-up evaluation; and
(f) able to provide written informed consent.

Participants will not be restricted from using any pharmaceutical agents or Chinese herbal medicines for treating either their facial paralysis or any other medical condition

### 2.5 Exclusion criteria

Participants with any of the following conditions will be excluded: uncontrolled hypertension; diabetes mellitus requiring insulin injection; other neurological diseases; multiple cranial nerve palsies; pregnancy and lactation. Participants with recurrent facial palsy or who have a pre-existing facial deformity, contracture, synkinesis, or spasm for whatever reason will be excluded. Study exclusion will also apply to the following participants: a surgical history for facial palsy such as facial nerve decompression, reconstruction of the facial nerve or muscle within the previous 3 months of study enrollment; oral ingestion of steroids or antiviral drugs (acyclovir, valaciclovir, famciclovir, or ganciclovir) within the previous 3 months of study enrollment; receipt of acupuncture, moxibustion, or massage therapy within the previous month of study enrollment. All types of physical therapy including electrical stimulation, TENS and facial exercises within 1 month. The researchers will exclude any participants considered to be inappropriate for the study.

### 2.6 Primary outcome

The primary outcome measurement will be the change from baseline at 6 weeks in FDI scores on the social subscale.^[18]^ The FDI scoring system consists of 2 sections, a physical score, and social score. Each section consists of 5 multiple choice questions relating to either physical or social issues that occurred in the previous month. The FDI has shown good reliability and validity.^[18]^ FDI score evaluations will be performed on the first visit, the 9th and 18th visits, and will be compared with baseline scores. As far as we are aware, no traditional Chinese version of the FDI exists. We have therefore translated and proofread the English version into traditional Chinese.^[18]^

### 2.7 Secondary outcome

The secondary outcomes will consist of changes from baseline at 6 weeks in FDI physical subscale scores, the H-B, and S-B facial nerve grading system and stiffness scale. The H-B and S-B systems have shown good reliability.^[19]^ Both grading systems will be performed on the first visit, the 9th and 18th visits, and will be compared with baseline scores. The stiffness scale will be evaluated in each session and compared with baseline values. On a scale of 1 to 5, the participant will be asked to select the number that best represents his/her level of stiffness (where 1 = no stiffness to 5 = very stiff).^[20]^

### 2.8 Randomization and blinding

At visit 1, after undergoing screening for inclusion/exclusion criteria and baseline assessments, participants will be randomized to either the experimental (real LAT treatment) or the control (sham LAT treatment) group using a block randomization method^[21]^ and a randomization sequence created by Excel 2016 (Microsoft Office) before study commencement. The study groups will have 16 participants each. The balanced sample size will be ensured by dividing the subjects into even blocks of 2, 4, 6, or 8. Randomization will be conducted independently within each block; all 32 subjects will be divided into 4 blocks. For each block, 8 subjects will be randomly allocated into the intervention group or sham control group using a 1:1 ratio, irrespective of gender. Only the research assistant of this trial, who is responsible for this procedure and is not involved in the treatment, assessment, or data analysis, will know the randomization sequence. The participants, the investigators, the assessor, and the biostatistician will all be blinded to the allocation throughout the study period; the study researchers will remain blind to treatment allocation until the data are analyzed. participants will be allowed at any time point to withdraw from the study. In case that withdraw occurs and the participant will be interested to know group allocation; the study research assistant would provide the relevant information for the participant which include, participant's treatment assignment and outcome data.

### 2.9 Laser parameters

This study will use the Handylaser Trion laser manufactured by RJ Laser, Germany. It will be used for 40 seconds to deliver 3J of energy as a pulsed wave (Noiger E) at each acupoint on the affected side. For distal points in the limbs, the laser will be used for 80 seconds at each acupoint to deliver 6J of energy as a pulsed wave (Noiger B) (Table 3). Protective goggles will be used by both the investigator and the patient as a form of protection from any laser damage to visual perception during irradiation. LAT represents a non-invasive, pain-free method of treatment and previous studies^[12–14]^ using LAT in Bell's palsy or other conditions^[22]^ have not reported any side effects caused by such treatment. Nevertheless, all adverse events occurring as a result of LAT will be recorded and monitored by the research investigator. Our study follows the World Association of Laser Therapy (WALT) guidelines for the LLLT intervention.^[23]^

**Table 3:**
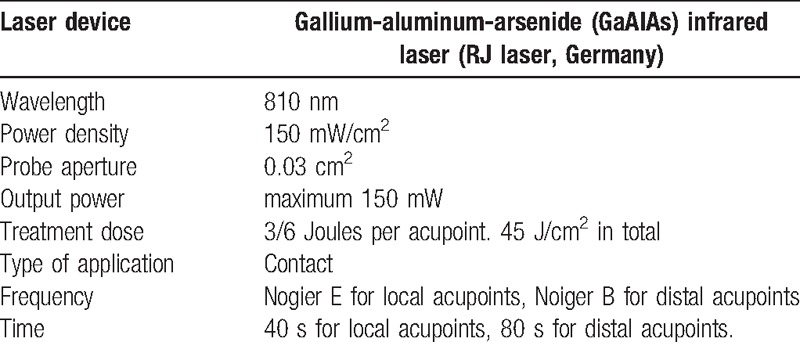
Parameters of the laser device in our study.

### 2.10 Data collection

Personal information for each participant, including the written informed consent form, the FDI questionnaire, H-B S-B grading systems, and the S-B stiffness scale, will be managed by the research assistant and stored in a private place. All data will be scanned, converted into PDF files and stored in a USB drive with a valid password. Our study will not use the patient's name as a means of identification. The research assistant responsible for randomization will assign a study identification number to each participant. Confidentiality and privacy protection of the patient's personal information will be maintained throughout the entire clinical trial procedure. The data will be stored for 3 years after the study completion. While the study will be conducted, every 6 months an auditing trial conduct, which includes study procedure and data collection, will be executed by one of the research members who is not involved in treatment, assessment, or data analysis.

### 2.11 Data monitoring

LAT represents a non-invasive method of treatment, thus, data monitoring committee (DMC) is not needed.

### 2.12 Power analysis

The number of patients required for our pilot study is based on FDI data from a study in which the mean FDI score in the intervention group before the treatment was 68.6 with a standard deviation of 14.1, while the mean FDI score after the treatment was 80.7 with a standard deviation of 12.2.^[20]^ The sample size was based on a power of 80% (beta 0.2), statistical significance (alpha 0.05) of 95% (*P* = .05) and a dropout rate of 20%. Thus, this study will require 32 patients.

### 2.13 Statistical analysis

Data analysis will be performed using SAS for Microsoft Windows, Version 9.4. The sample size and power calculations will be performed with G-power 3.0.10 for Microsoft Windows. Frequency and distribution will be assessed for categorical and numerical variable respectively. Wilcoxon signed rank test and Friedman test will be used to compare the FDI H-B, S-B scores and stiffness scale within each group as non-parametric analysis. Paired-T and ANCOVA will be used to compare the difference of correlated measurements within the subjects when distribution assumption fits. Generalized estimating equation (GEE) will be further applied to compare between measurements that will be taken at the first visit (baseline), after the 9th visit and 18th visit of the 2 groups and further multiple variable regression will be assessed in order to control the related risk factors.

### 2.14 Ethics

The study protocol has been approved by the Research Ethics Committee of the China Medical University Hospital, Taichung, Taiwan (Protocol ID: CMUH107-REC1-030) in accordance with the Declaration of Helsinki. The study is registered on ClinicalTrials.gov (Protocol ID: NCT03592797). A written informed consent form will be obtained from each participant. All participants will be allowed at any time point to withdraw from the study.

## 3 Discussion

We believe that this pilot study is the first-ever investigation into the use of LAT for the management of chronic Bell's palsy. Recent trials have described positive outcomes using acupuncture for chronic Bell's palsy^[8]^ and LLLT trials have shown good results in the treatment of acute Bell's palsy.^[13,14]^ This study aims to determine if LAT (delivered as gallium-aluminum-arsenide [GaAIA], infrared, 810 nm, 150 mw/cm^2^, 3/6 Joules per local/distal acupuncture point, applied for 40/80 seconds) improves physical and/or social functioning in patients experiencing inadequate recovery from chronic Bell's palsy.

In Asia, Bell's palsy is commonly treated with acupuncture in outpatient clinics.^[24]^ In a survey conducted in Taiwan from 2002 through 2011, the use of acupuncture for Diseases of the Nervous System and Sense Organs ranked fourth among adult patients.^[25]^ In 1996, the World Health Organization published a comprehensive review on acupuncture controlled clinical trials^[26]^ and outlined 64 symptoms treatable by acupuncture; Bell's palsy was included. However, all of the included clinical trials in that review focused on acute Bell's palsy. Clinical evidence is needed for the efficacy of acupuncture in chronic Bell's palsy.

LAT represents a non-invasive, pain-free method of treatment that has largely been investigated for its efficacy in the relief of pain-related conditions. Systematic reviews have discussed evidence in support of LAT.^[22,27]^ Importantly, when using laser acupuncture, the type of disease and laser parameters can greatly influence treatment outcomes,^[28]^ so must be taken into account; these variables include the wavelength, power output, and duration of irradiation. Notably, our study will differ from previous studies in that none of them used acupoints as target sites. We anticipate that our results will clarify how varying laser parameters may influence the clinical outcomes in the treatment of patients with the peripheral type of Bell's palsy. The results of this study are expected to indicate whether or not LAT is an effective therapy for patients experiencing inadequate recovery from Bell's palsy.

### 3.1 Trail status

This clinical trial is currently recruiting patients. Recruitment procedure began on May 2018. We expect to complete this procedure on May 2020.

### 3.2 Appendices

A copy of the written consent form is available from the Editor-in-Chief of this journal.

## Acknowledgments

The authors would like to thank Mrs Wen-Chi Lu, study's research assistant, for her kind assistance in the randomization and blinding procedure and recruiting the study participants. The authors also thank Ms Iona MacDonald for the English proofreading for this manuscript.

## Author contributions

GT is the first author responsible for preparing the manuscript; YCL is the principal investigator and corresponding author; LWL is the research investigator, GT is the second investigator. YCL and HYL are the outcome assessors. YCL and GT conceived the study; GT, YCL, CHTS, CHT, and YHC are responsible for the study design. WCH is responsible for statistical analyses; GT will collate the data and prepared the manuscript; HPN provided critical revision of the manuscript and the FDI Chinese version. All authors reviewed and approved the final version of the manuscript.

**Conceptualization:** Gil Ton, Yu-Chen Lee.

**Formal analysis:** Wen-Chao Ho.

**Investigation:** Li-Wen Lee.

**Methodology:** Gil Ton, Yi- Hung Chen, Cheng-Hao Tu, Chun-Hung Tseng.

**Resources:** Yi- Hung Chen, Yu-Chen Lee.

**Supervision:** Yu-Chen Lee.

**Validation:** Hsien-Yin Liao, Yu-Chen Lee.

**Visualization:** Gil Ton, Yu-Chen Lee.

**Writing – original draft:** Gil Ton.

**Writing – review & editing:** Hui-Ping Ng.

Gil Ton orcid: 0000-0002-2065-797X.
